# Cooperative population coding facilitates efficient sound-source separability by adaptation to input statistics

**DOI:** 10.1371/journal.pbio.3000150

**Published:** 2019-07-29

**Authors:** Helge Gleiss, Jörg Encke, Andrea Lingner, Todd R. Jennings, Sonja Brosel, Lars Kunz, Benedikt Grothe, Michael Pecka

**Affiliations:** 1 Division of Neurobiology, Department of Biology II, Ludwig-Maximilians-Universitaet Muenchen, Martinsried, Germany; 2 Chair of Bio-Inspired Information Processing, Department of Electrical and Computer Engineering, Technical University of Munich, Garching, Germany; University College London, UNITED KINGDOM

## Abstract

Our sensory environment changes constantly. Accordingly, neural systems continually adapt to the concurrent stimulus statistics to remain sensitive over a wide range of conditions. Such dynamic range adaptation (DRA) is assumed to increase both the effectiveness of the neuronal code and perceptual sensitivity. However, direct demonstrations of DRA-based efficient neuronal processing that also produces perceptual benefits are lacking. Here, we investigated the impact of DRA on spatial coding in the rodent brain and the perception of human listeners. Complex spatial stimulation with dynamically changing source locations elicited prominent DRA already on the initial spatial processing stage, the Lateral Superior Olive (LSO) of gerbils. Surprisingly, on the level of individual neurons, DRA diminished spatial tuning because of large response variability across trials. However, when considering single-trial population averages of multiple neurons, DRA enhanced the coding efficiency specifically for the concurrently most probable source locations. Intrinsic LSO population imaging of energy consumption combined with pharmacology revealed that a slow-acting LSO gain-control mechanism distributes activity across a group of neurons during DRA, thereby enhancing population coding efficiency. Strikingly, such “efficient cooperative coding” also improved neuronal source separability specifically for the locations that were most likely to occur. These location-specific enhancements in neuronal coding were paralleled by human listeners exhibiting a selective improvement in spatial resolution. We conclude that, contrary to canonical models of sensory encoding, the primary motive of early spatial processing is efficiency optimization of neural populations for enhanced source separability in the concurrent environment.

## Abstract 2

The efficient coding hypothesis suggests that sensory processing adapts to the stimulus statistics to maximize information while minimizing energetic costs. This study finds that an auditory spatial processing circuit distributes activity across neurons to enhance processing efficiency, focally improving spatial resolution both in neurons and in human listeners.

## Introduction

**Fig 1:**
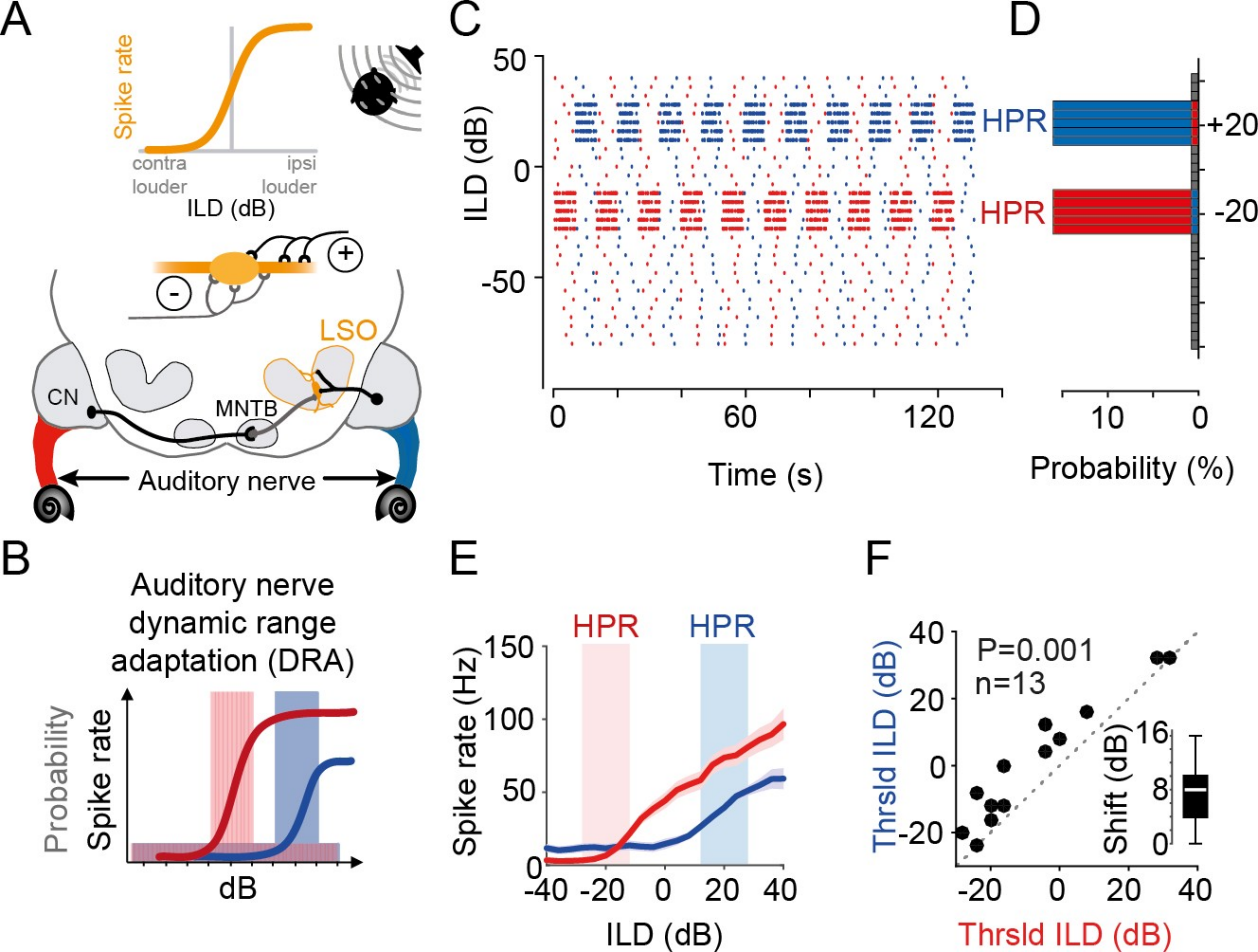
Statistic-dependent coding of spatial cues in LSO. (A) Upper panel: LSO neurons respond with increasing higher spike rates to increasingly more ipsilateral sound-source positions because these positions generate more “positive” ILDs (ILDs that favor the excitatory ear). Lower panel: circuit diagram of the inputs to the LSO. LSO neurons receive excitatory input from the ipsilateral ear via the CN and inhibitory inputs from the contralateral ear via the MNTB. (B) Non-unimodal probability distributions of monaural stimulus intensity already cause pronounced DRA on the level of the ANFs [16]. Thus, for large ILDs, the ANFs in the left and right ear will adapt to different intensity levels (as indicated by color-coded ANFs in A). Colored shaded areas illustrate respective HPRs of stimulus value occurrence. See also (C) and (D). (C) Illustration of the temporal sequence of ILDs in the two HPR conditions (centered on −20 dB ILD, shown in red, or +20 dB ILD, shown in blue) that were used to test the effect of complex stimulation on ILD coding. Note that the sequence changed for each of the 10 iterations of each HPR epoch. (D) Probability histogram of the sequences shown in (C). 80% of stimuli had ILDs centered on either −20dB ILD ± 8 dB (red) or +20 dB ILD ± 8 dB (blue). (E) Representative example of DRA in response to the change in HPR condition in a single LSO neuron. The ILD response function for this neuron was substantially different between the −20 dB ILD ± 8 dB (red) or +20 dB ILD ± 8 dB (blue) condition. Given are mean response rates (solid lines) and SEM (shaded area). (F) Threshold ILDs (minimal ILD that significantly differed from baseline; see Materials and methods) shift significantly by altering the HPR condition (*P* = 0.001, paired Wilcoxon signed-rank test, *n* = 13 neurons). Inset shows median shift (8 dB, white bar), with IQR given by the box edges. Whiskers extend to overall data range. Underlying data can be found in S1 Data. See also S1 Fig. ANF, auditory nerve fiber; CN, Cochlear Nucleus; DRA, dynamic range adaptation; HPR, high-probability region; ILD, interaural level difference; IQR, interquartile range; LSO, Lateral Superior Olive; MNTB, Medial Nucleus of the Trapezoid Body; Thrsld, threshold.

Our ability to distinguish individual objects in complex and dynamic environments is a fundamental brain function [1,2]. Conversely, the functional requirements of sensory systems are shaped by the physical properties of the outside world: only if the neural sensitivity matches the current statistics of the sensory inputs will the coding of relevant stimulus features be both informative and energetically efficient and consequently evolutionarily viable. Because realistic complex environments exhibit highly nonuniform occurrence probabilities of stimulus cues [3,4], sensory neurons adapt their action potential (“spike”) responses according to the probability of concurrent stimulus properties. This “dynamic range adaptation” (DRA) is thought to render neuronal firing maximally sensitive to changes in the stimulus range that is most likely to occur (Fig 1) [5–7] while keeping activity rates low. Consequently, DRA to stimulus statistics is believed to reflect a neuronal adjustment to optimize stimulus encoding efficacy while simultaneously mediating improved perceptional resolution in the relevant cue range. However, direct demonstrations of DRA-based neuronal coding that causes both increased neuronal efficiency and the resulting perceptual benefits are lacking.

In the auditory system, rapid (subsecond) DRA to stimulus statistics has been revealed on multiple processing levels from primary auditory cortex [8–11] to the midbrain [12–15] and even brainstem (Fig 1A). Specifically, DRA is prominently exhibited already by auditory nerve fibers (ANFs) [16,17] (Fig 1B), which consequently should affect the processing of all downstream centers but might potentially be most crucial for spatial computations.

To infer the location of a sound source, brainstem neurons of the Lateral Superior Olive (LSO) compare the difference in sound level at the two ears (interaural level difference [ILD]) that is generated by a location-specific sound-attenuating effect of the head. LSO neurons respond according to the relative strength of excitatory and inhibitory inputs from the ipsi- and contralateral ear, respectively (Fig 1A). The ensuing sigmoidal ILD response functions (average action potential rate as a function of ILD) are regarded as representing the neuronal basis of auditory space encoding based on intensity difference cues [18] (Fig 1A, top, in addition to timing cues not dealt with in the present study). Specifically, individual source locations are thought to be mapped onto a specific spiking activity pattern of precisely tuned neurons or neuronal populations [19–22]. Yet, the nature of this spatial code and its readout is still a matter of debate [23–26]. Historically, studies have argued in favor of a labeled-line coding strategy of auditory space (or a mix of strategies), in which small differences in the average spike-rate tuning of individual, identifiable neurons or subpopulation contribute to sound-source localization [27–29]. Yet, the majority of recent studies have concluded that sound-source locations are initially encoded by the specific relative spike rate of two oppositely tuned hemispheric populations of spatially sensitive neurons (for review, see [18]). This “two-channel hemispheric coding strategy” is motivated by the fact that the vast majority of neurons in each brainstem hemisphere are broadly and similarly tuned, thus providing redundant information about sound-source locations (reviewed in [30]). The reasons for such apparently inefficient coding of space are, however, unknown. In either case, conclusions about spatial coding were derived from examining average neuronal firing rates in response to multiple repetitions of a stimulus set with uniform probability distributions of spatial cues (e.g., each ILD was equally likely to occur). Consequently, these traditional approaches neglected that under more natural conditions, DRA (of ANFs or later stages) might crucially alter the nature of the neuronal code and/or its perceptual consequences.

First, the fact that sound-source positions far to the left or right will result in distinctly different sound levels at the two ears (i.e., a large ILD) consequently should evoke DRA to different (monaural) stimulus levels for the ANFs in the left and right ear. Yet, it remains to be tested how such differential monaural DRA impacts the detection and representation of ILDs in the LSO.

Interestingly, an earlier study from the midbrain had reported that the sensitivity to ILDs undergoes DRA as a function of the spatial statistics [15]. These data thus suggest that spatial processing downstream of the LSO may adapt to accentuate relative differences in sound-source positions in complex environments. We previously also identified an activity-dependent LSO gain-control mechanism [31] that might additionally influence the response to ILDs based on stimulation history [32]. It follows that the extraction of ILDs, and consequently the primary representation of auditory space, already might not be as rigid as traditionally assumed. More fundamentally, since DRA to absolute sound level is already prominently exhibited by ANFs [16,17], it is unclear to which extent the observed adaptation to ILDs at later stages are the direct result of adaptation at either ear alone.

Second, the auditory pathway—like all sensory systems—must detect and code the relevant stimulus properties from only a single stimulus occurrence. The nature of response distributions to such a single instance of, e.g., an ILD in a neuron population might be very different compared to the response distribution of a single neuron to this ILD averaged across trials and consequently could result in different coding regimes. Since the extent of DRA can vary considerably across cells [10,16], such differences in response distributions might be particularly evident in complex acoustic environments.

Third, neuronal adaptations such as DRA to increase computational efficiency supposedly also entail a behavioral improvement within the concurrent environmental conditions. Hence, important insight into how the brain encodes auditory space under complex conditions might be gained by investigating the perceptual impact of DRA [22]. Yet, while perceptual changes due to neuronal adaptation to stimulus statistics have been reported [15,33], demonstrations of how these changes are linked to improving neuronal efficiency are missing.

To answer these questions, we studied the effects of spatially complex stimulation on ILD processing in the LSO of gerbils and on the perception of human listeners. We extended a well-established monaural stimulus paradigm for studying DRA [10,12,16,17] by generating a binaural version of these stimuli to specifically test how spatial coding in the LSO is affected by DRA on its monaural inputs. Our stimulus paradigm resulted in rapidly changing ILDs that switched periodically between favoring either the left or right azimuthal space (Fig 1C and 1D), as can be experienced in noisy environments [34]. In response to these spatially dynamic stimuli, we observed prominent DRA in LSO neurons, which demonstrate a lack of absolute encoding of space by average neuronal firing rate. Surprisingly, DRA in single neurons resulted in large response variability to a given ILD across trials. However, we find that when considering single-instance population coding, DRA maximized the efficiency of neuronal separability for specifically those ILDs that were most likely to occur in the concurrent statistical environment (high-probability region [HPR], Fig 1C and 1D). These enhancements in neuronal coding were paralleled by human listeners exhibiting a selective improvement in just-noticeable differences (JNDs) for ILDs in the hemisphere of the respective HPR. Intrinsic LSO population imaging of energy consumption and a simple LSO model further explained that a slow-acting gain-control mechanism enhances the population efficiency by distributing activity across a group of neurons during DRA. We conclude that already on the primary detector level, the processing of ILDs is not tuned towards a representation of locations in space but optimizes efficient sound-source separation in the concurrent acoustic environment by instantaneous population coding of ILDs.

## Results

### LSO neurons exhibit DRA to stimulus statistics

To explore the role of DRA on spatial coding in complex environments, we designed a stimulus paradigm with constantly varying ILDs in the context of two related but statistically distinct listening conditions. We used continuous broadband noise (identical on the two ears) and changed the ILD every 50 ms, with ILD values drawn from one of two nonuniform distributions. The two distributions covered an identical range of ILDs but favored predominately (80% of time) either the ipsi- or contralateral ear (ILDs of +20 dB ± 8 dB and −20 dB ± 8 dB, named the +20 dB HPR and −20 dB HPR, respectively; Fig 1C and Materials and methods). This way, we simulated dynamic spatial environments with dominant sound sources located either left or right of midline (Fig 1D). The two conditions switched periodically (Fig 1C: 1 run consisted of 19 switches every approximately 6 s; the sequence of ILDs was different for each switch but identical across repetitions; 3 runs were recorded for each cell). To assess to what extent changes in stimulus statistics on the two ears alter the neuronal detection and encoding of ILDs, we first carried out extracellular recordings from single neurons in the LSO of anesthetized gerbils while presenting the stimuli via calibrated earphones (see Materials and methods).

Following previous studies of DRA [12,14–16], we first assessed the average neuronal spike rates (calculated across occurrences of each ILD) separately for −20 dB and the +20 dB HPR conditions. We observed that the resulting ILD response functions differed between the two conditions (a single-neuron example is shown in Fig 1E). Specifically, a clear shift of the ILD-spike–rate functions was observable that entailed a change in the average spike rate in the respective HPRs (red and blue areas in Fig 1E and throughout). To quantify these shifts, which appeared highly reminiscent of DRA to accommodate the change in the range of overrepresented ILDs, we computed the minimal ILD that triggered significant spiking (“threshold ILD”; see Materials and methods) in the respective condition for each neuron. Threshold ILDs significantly increased when switching from the −20 dB to the +20 dB HPR (Fig 1F, *n* = 13 neurons, *P* = 0.001, paired Wilcoxon signed-rank test). For the population, the median shift in threshold ILD between the two conditions was 8 dB (interquartile range [IQR] 6 dB; Fig 1F inset). To further characterize the extent and specificity of the DRA, we also generated two additional ILD distributions (*n* = 18 neurons and *n* = 19 neurons), as well as a monaural condition (stimulation only on the ipsilateral ear, *n* = 11 neurons). These additional conditions confirmed that the observed shifts in threshold ILDs were dependent on the concurrent input statistics (S1 Fig). This presence of DRA-related shifts in ILD functions in the LSO directly demonstrates a lack of absolute encoding of sound-source locations by the average neuronal firing rate already on the level of cue detection.

### DRA optimizes single-observation population coding

**Fig 2:**
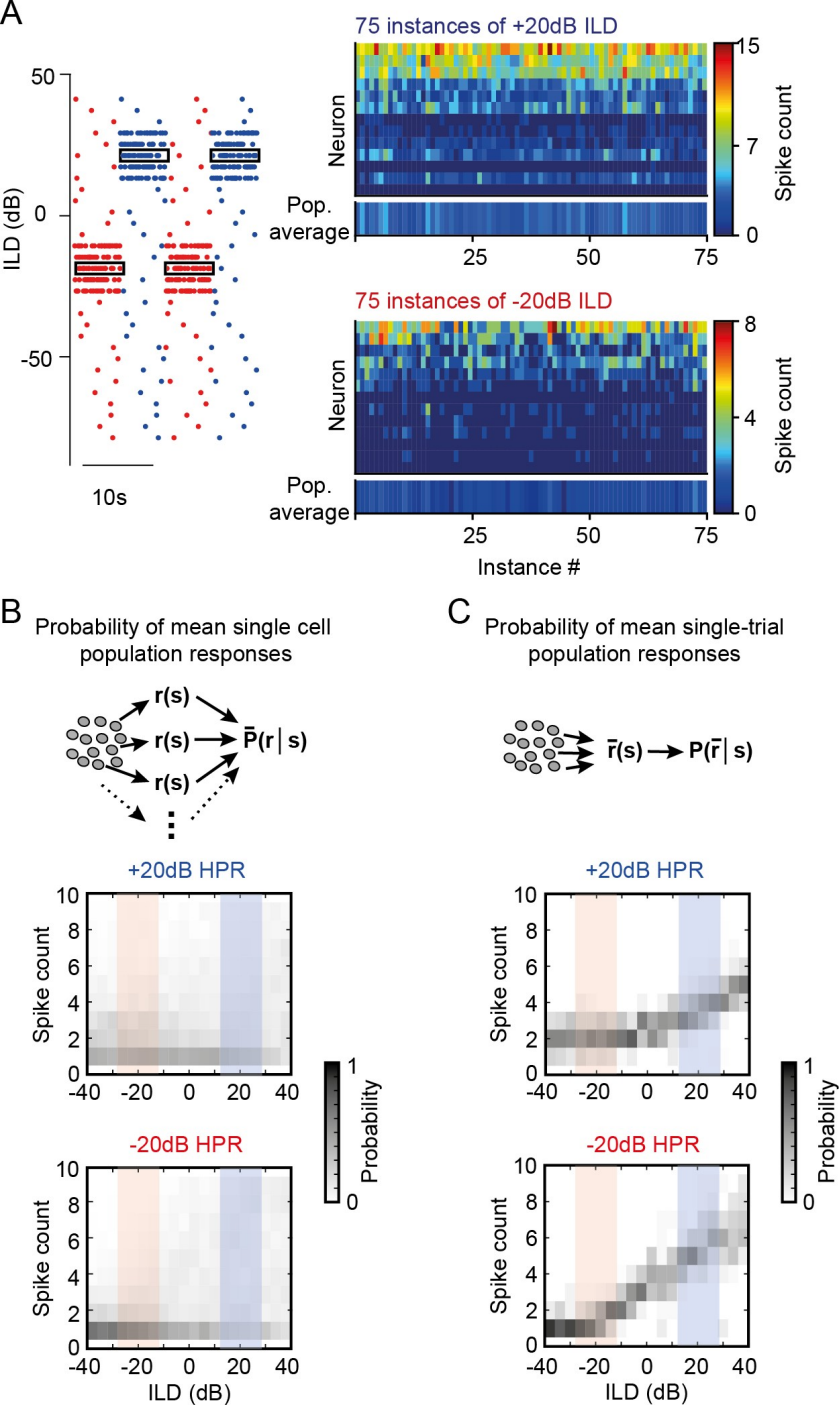
Statistic-dependent DRA results in sparse and selective ILD coding for single-instance population averages. (A) While response probabilities were low overall, the variability in spike counts to repetitive instances of the same ILD in individual neurons was high. Shown are spike counts of all neurons in response to 75 instances of −20 dB ILD (lower right-hand panel) or +20 dB ILD (upper right-hand panel). Bottom line in each panel shows mean responses for each ILD instance. (B and C) Mean population spike-count probability density functions of all neurons were constructed based on the pooled response of all single-neuron responses (B) and pseudopopulation mean responses at each instance of ILD occurrence (C). Only the latter resulted in informative ILD tuning (see also Fig 3 and S2 Fig). Underlying data can be found in S1 Data. DRA, dynamic range adaptation; HPR, high-probability region; ILD, interaural level difference; Pop., population.

So far, we followed previous studies of DRA in the auditory system [12,14–16] and evaluated the spatial sensitivity of individual LSO neurons by their average spike rate given the repeated presentation of each ILD. However, in reality, processing must be able to compute the location of a sound source from observation of a single instance of the stimulus. Therefore, we next focused on the direct response by each neuron to each occurrence of a particular ILD. Examining individual spike counts for 75 recurrent instances of +20 dB and −20 dB ILDs in the respective HPR condition revealed two interesting findings (Fig 2A).

First, responses of most LSO neurons for ILDs from the concurrent HPR were very sparse (median spike count and IQR: −20 dB HPR, 0.92 and 0.46 spikes; +20 dB HPR, 3.15 and 0.85 spikes). Second, a high response variability, as indicated by the large IQR, was observable for all ILDs: spike counts varied considerably between repeated instances of the same ILD (trial-wise median Pearson’s correlation coefficient and IQR: 0.62 and 0.26, S2 Fig), and spike-triggered average analysis showed no systematic relationship between ILD sequences and their likelihood to trigger a spike (S2 Fig). Crucially, this lack of consistent responses to ILDs with complex probability statistics resulted in a very limited modulation of the average spiking probabilities in either HPR condition, i.e., the probability to observe a particular mean average spike count across the sample population was very similar for all ILDs (Fig 2B).

**Fig 3:**
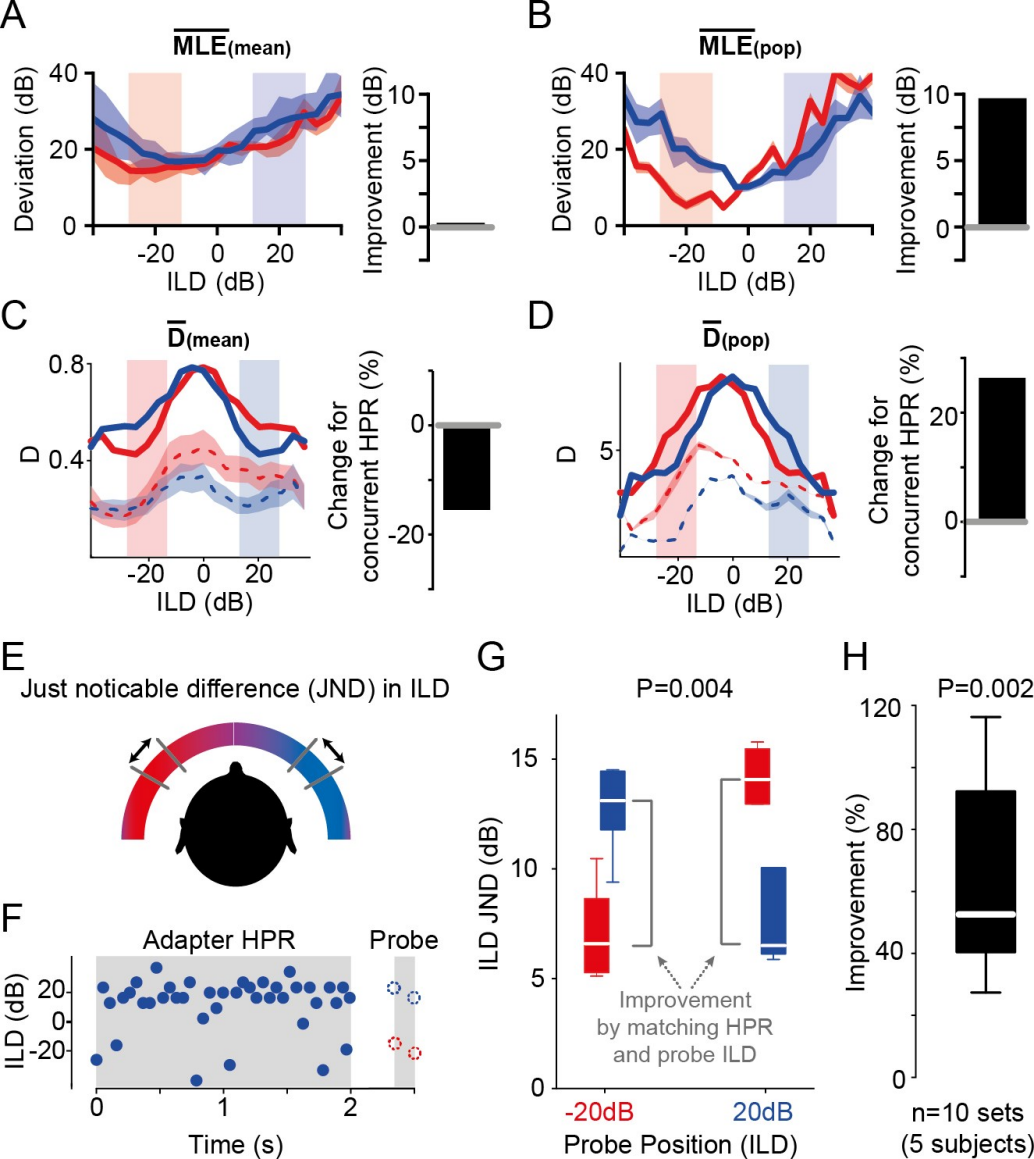
Single-instance population averaging predicts human spatial perception. (A) Average MLE(mean) exhibited no apparent advantage of DRA for decoding ILDs (improvement = 0%, right-hand panel), as the tuning function obtained from the −20 dB HPR condition was overall better in estimating the ILDs compared to those obtained from the +20 dB HPR condition. Solid lines show the mean deviations, and shaded area represents SEM. (B) Average MLE(pop) showed a 9-dB improvement (calculated as the difference between the red and blue functions within the HPRs) for decoding ILDs from the concurrent HPR. Conventions as in (A). (C) Average D(mean) across all cells (*n* = 13) decreased for the concurrent HPR, suggesting a relative worsening of the ability to distinguish adjacent ILDs by −15.5% (right-hand panel). Solid lines represent D, including the hypothetical second hemispheric response. Mean data from a single hemisphere are given by dashed lines (shaded area represents SEM). (D) Average D(pop) of the recorded LSO neurons (*n* = 13) increased for ILDs from the concurrent HPR by 27.1% (right-hand panel). Solid lines represent D(pop), including the hypothetical second hemispheric response. Mean data from a single hemisphere are given by dashed lines (shaded area represents SEM). (E) and (F) The effect of DRA to HPRs on ILD JND of human subjects were measured with stimuli presented over headphones. Presentation of an adapter sound consisting of a 2-s snippet of one of the two HPR stimuli was followed by two noise probes (50 ms each) for JND measurement. The ILDs of the probe tones were centered on −20 dB ILD or +20 dB ILD and hence either matched or mismatched the HPR of the preceding adapter. (G) Single-subject example of the influence of the HPR on ILD JND. Colocation (i.e., matching) of the adapter HPR and probe sound position led to significant improvement of the ILD JND (*P* = 0.004, Friedman test, *n* = 12 trials each). (H) The average improvement of colocation by adapter HPR and probe sound position across listeners was 52.6% (*P* = 0.002, unpaired Wilcoxon signed-rank test, *n* = 10 sets from 5 subjects, conventions as in inset in Fig 1F). Underlying data can be found in S1 Data. DRA, dynamic range adaptation; HPR, high-probability region; ILD, interaural level difference; JND, just-noticeable difference; MLE, Maximum Likelihood Estimation; pop, population.

In contrast, however, more specific ILD population tuning emerged from our data set when considering the pseudopopulation response for a single occurrence of a particular ILD (i.e., averaging across a column in Fig 2A and 2C; compare also bottom lines in right-hand panels of Fig 2A; note that neurons were recorded sequentially). To determine how these different population tunings of mean single-cell population responses and single-instance pseudopopulation responses impact the decoding accuracy of ILDs, we performed a Maximum Likelihood Estimation (MLE; see Materials and methods) for both methods. In short, MLE approximates which ILD is most likely to have occurred given the observation of a particular spike count. The estimates differed in two important ways when either considering the mean single-cell population responses (MLE[mean]) or the single-instance pseudopopulation response (MLE[pop]) (Fig 3A and 3B): First, the estimated deviation from the true ILD values were much larger for the MLE(mean) (minimal deviations: −20 dB HPR, 14.3 dB at −24 dB; +20 dB HPR, 16.8 dB at −16 dB) compared to MLE(pop) (minimal deviations: −20 dB HPR, 4.7 dB at −20 dB; +20 dB HPR, 10.1 dB at 0 dB); i.e., the accuracy of MLE(pop) was higher. Secondly, the generally assumed advantageous effect of DRA, i.e., that DRA explicitly enhances the coding in the respective HPR, was evident for MLE(pop) but not MLE(mean) (Fig 3A and 3B; improvement of 9 dB and 0 dB, respectively).

Such relative enhancement in the neuronal precision of ILD estimation implies—but does not confirm—a relative improvement in the ability to resolve nearby sound locations. To quantify the impact of the differences in population tuning (Fig 2B and 2C) on resolution directly, we next determined the informational content of each neurons’ response towards the ability to distinguish adjacent ILDs. We followed previous studies on ILD coding [15,35] and calculated the standard separation (“D”) [36], which quantifies the separability of adjacent ILDs based on the ratio of slope steepness and response variability. We first calculated D(mean), i.e., the mean D across neurons, which is derived from averaging the D-ILD functions of each single neuron. Since the monaural stimuli that we presented in the −20 dB and +20 dB HPR epochs were mirror-symmetric to each other, the responses that we recorded in the LSO of one hemisphere during each HPR epoch can be assumed to reflect the responses to the other HPR epoch in the LSO in the other brain hemisphere (compare [12,15]). In other words, the LSO on each side of the brain would provide complementary spatial information for each HPR condition towards D. We therefore summed the D-ILD functions of each condition with the mirror-image of the function of the other condition (Fig 3C; dashed lines indicate single-hemisphere data, solid line represents sum of both hemispheres). In remarkable contrast to previous midbrain studies [12,15), we found that the average neuronal separability was not enhanced by the DRA but actually considerably lower for the ILDs from the respective HPRs (Fig 3C; change for concurrent HPR: −15%, compare red and blue lines in respective HPRs in left panel). Thus, D(mean) would predict a worsening of ILD resolution by the observed DRA to spatial stimulus statistics. In contrast, when D is calculated based on the mean spike count of all neurons to each instance of an ILD (D[pop]), a specificity of separability for the ILDs of the concurrent hemisphere, including the HPR, becomes evident (Fig 3D; change for concurrent HPR: +27%). Similarly, a distinct benefit of D(pop) over D(mean) was also observed for the two additional binaural stimulus paradigms we tested (S1 Fig).

### Human spatial resolution improves specifically for HPR ILDs

To directly test whether the increased performance as predicted by analyzing MLE(pop) and D(pop) also results in an improved ability to resolve sound-source locations, we performed a spatial separability test with human listeners via calibrated headphones (Fig 3E). The subjects (*N* = 5) were presented with a 2-s–long snippet of the same stimulus used in the electrophysiological experiments, taken alternatively from the +20 dB and −20 dB ILD HPR condition (only +20 dB is illustrated in Fig 3F). Shortly after (0.35 s) this adapting period, the listeners were presented with two probe ILDs (each consisting of 50 ms broadband noise, spaced apart by 100 ms) and were asked to indicate which of the two was perceived more lateralized. Using an adaptive tracking paradigm (see Materials and methods), the difference in ILD between the two probe ILDs was systematically reduced to determine the JND in ILD for each subject. The probe ILDs were centered either on +20 dB ILD or −20 dB ILD (Fig 3F) to allow for deciphering the influence of matching and mismatching the adapter conditions. Specifically, the electrophysiological data suggested that JNDs should be enhanced for ILDs in the same hemisphere as the adapter HPR (Fig 3D). In agreement with this prediction made on the basis of D(pop), we observed a significant improvement in JND when probe center ILDs matched the hemispheric bias of the adapter (single-subject example in Fig 3G: −20 dB adapter and −20 dB probe, red, or +20 dB adapter and +20 dB probe, blue; *P* = 0.004, Friedman test). On average, JNDs of the five listeners improved by 52.6% (Fig 3H, IQR: 51.8%, *P* = 0.002, Wilcoxon signed-rank test). Thus, human JND performance is in close agreement with the neuronally derived MLE(pop) and D(pop), suggesting that DRA crucially affects both population coding and perception of ILDs in complex acoustic environments.

### Slow gain control maximizes efficiency

**Fig 4:**
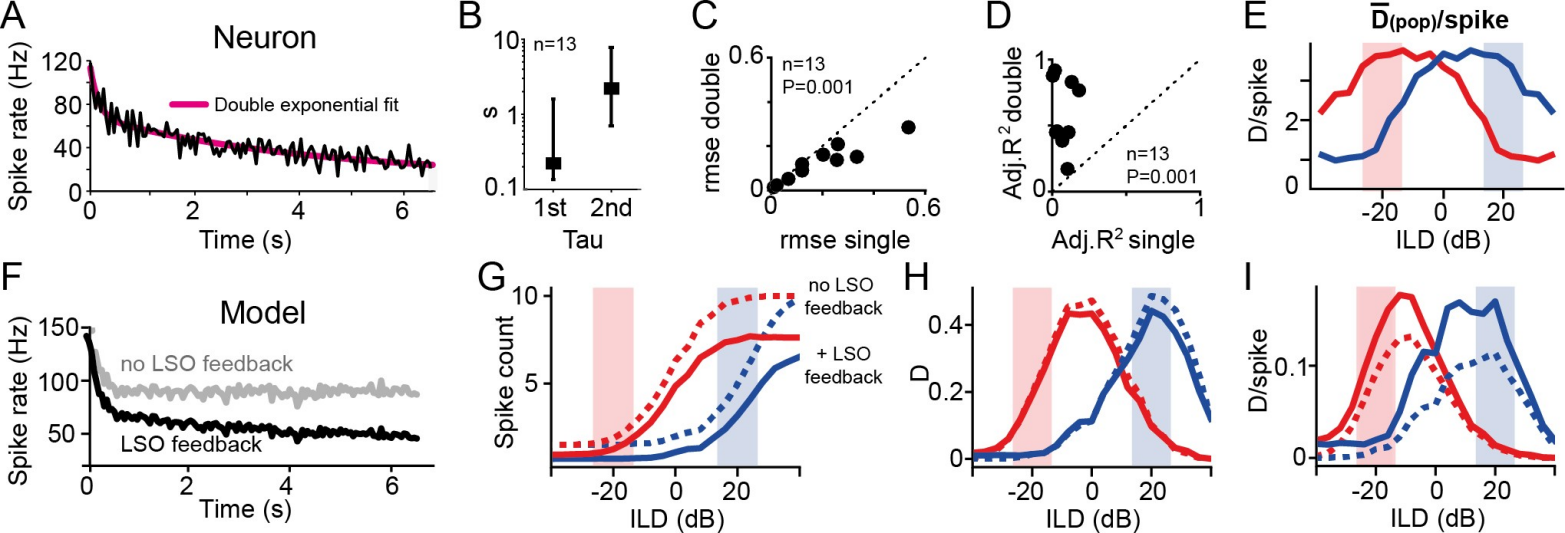
Slow rate adaptation increases population efficiency. (A) Evolution of rate adaptation is best explained by a double-exponential process. Shown is a single-neuron example (black) and double-exponential fit (magenta). Besides a rapid rate adaptation at the beginning of an epoch, a second, slow time constant was also present in gerbil LSO. (B) Average fast and slow time constants of DRA in the LSO from double-exponential fitting: median tau(first) = 0.22 s, IQR: 1.465 s; median tau (second) = 2.2 s, IQR: 7.7 s. (C and D) Inclusion of a second time constant was superior to single time constant fitting of rate adaptation: the rmses of fits decreased (C; *P* = 0.001, paired Student *t* test, *n* = 13 neurons) and adjusted R^2^-values increased (D; *P* = 0.001, paired Student *t* test, *n* = 13 neurons). (E) Efficiency of responses was measured by calculating D(pop)/spike, displaying high specificity for ILDs in the concurrent HPR. (F) A simple subtraction model of the LSO and DRA of its inputs can replicate the electrophysiological results only when including a binaural gain-control stage. Shown are adaptation time courses of the model (as for the neuron in A). Gray and black traces represent results excluding and including binaural negative feedback, respectively. (G, H, I) The model was able to qualitatively reproduce both the HPR-specific shifting effect of DRA in the LSO (G) and the HRP specificity of D (H) and D/spike (I). Model responses without and with slow negative feedback stage after binaural processing are shown by dashed and solid lines, respectively. Crucially, the presence of negative feedback resulted in stronger rate adaptation, which did not affect D but substantially increased efficiency by 55% (mean improvement within the two HPR regions). Underlying data can be found in S1 Data. DRA, dynamic range adaptation; HPR, high-probability region; ILD, interaural level difference; IQR, interquartile range; LSO, Lateral Superior Olive; rmse, root mean-squared error.

How could single-instance population responses in the LSO be optimized for separability in the concurrent HPR? Moreover, what effect might link high response variability of single neurons and highly informative population ILD coding? To gain insight into potential underlying mechanisms, we first analyzed the time course of DRA in LSO neurons. In accordance with DRA studies using similar stimulus statistics in other centers of the auditory system [12,14,16], we observed an exponential time course of rate adaptation (Fig 4A). Yet, in contrast to previous reports, we found that adaptation kinetics were best described not by a single but by two time constants (Fig 4B). Addition of the second time constant resulted in lower root mean-squared errors (rmses) of fits (median rmse_(double)_ = 0.149, median rmse_(single)_ = 0.255, *P* = 0.001, Wilcoxon signed-rank test; Fig 4C), and double-exponential fitting was significantly superior to single-exponential fits even after compensating for the unspecific benefit of an additional fitting parameter (Fig 4D; median adjusted R^2^_(double)_ = 0.45, median adjusted R^2^_(single)_ = 0.025, *P* = 0.001, Wilcoxon signed-rank test). We also fitted the time course of DRA using a power-law fit [37] and found that it was also superior to single-exponential fitting in explaining the observed time course (median rmse_(power)_ = 0.151, *P* = 0.001, Wilcoxon signed-rank test; S3 Fig). Indeed, rmse_(power)_ was comparable across cells, yet consistently slightly worse, to the double-exponential fitting (*P* = 0.02, Wilcoxon signed-rank test; S3 Fig). Moreover, double-exponential fitting had greater explanatory power towards the origin of the observed adaptation. Specifically, the shorter (first) time constants of the double-exponential fit (Fig 4B; median tau = 222.8 ms, IQR: 1.465 s) were similar to the kinetics reported for the auditory nerve [17], suggesting that monaural DRA upstream of the LSO contributed substantially to the ILD adaptations in the LSO. The second time constants were considerably slower and in the range of a few seconds (Fig 4B; median tau = 2.2 s, IQR: 7.7 s).

Such slow rate adaptation is consistent with previous reports of negative feedback loops in LSO neurons: the inhibitory transmitter *gamma*-aminobutyric acid (GABA) is released in an activity-dependent manner into the extracellular matrix and thereby suppresses presynaptic inputs in the vicinity via slow-acting GABA-B receptors [31]. Hence, DRA in the LSO might be considerably influenced by a slow binaural gain control for ILD coding during complex stimulation. It has been suggested that such slow negative feedback serves to increase the efficiency of population coding [38,39]: because spiking is energetically costly, the efficiency of a neuronal representation depends on the informational content of a spiking response relative to the number of spikes that conveyed this information [40,41]. To quantify neuronal population efficiency directly, we calculated the average D transmitted per spike for the instantaneous hemispheric average (D[pop]/spike). This analysis revealed a hemispheric specificity of response efficiency for the concurrent spatial conditions (Fig 4E). Thus, the slow gain-control mechanism associated with the second time constant of DRA that we found might serve to maximize the efficiency of neuronal processing within the hemisphere of the HPR. To investigate this potential role of slow gain control on ILD coding in more detail, we generated a simple model of the LSO based on existing models of DRA. Specifically, we extended an existing auditory nerve model that included both threshold and gain adaptation [17] by adding a binaural subtraction stage to reflect LSO processing (S3 Fig). As expected, this simple model exhibited clear DRA in response to the binaural HPR stimuli (Fig 4G, dotted lines, and S1 Fig), demonstrating that the HRP-specific shifts in ILD sensitivity are predominately caused by the oppositional monaural DRA of the LSO inputs. Likewise, the model was also able to reproduce the nature and extent of DRA to the two additional binaural stimulus paradigms that we tested (S1 Fig and S3 Fig).

However, since this version of the model lacked a binaural gain-control stage, it captured only the fast time course of rate adaptation and quickly reached a steady-state spike rate (<1 s, Fig 4F, gray trace). To account for the second, slow adaptation component in the neuronal data, we included an additional slow negative feedback stage after binaural comparison in the model (S3 Fig). This modification resulted in a close match in the dynamics of rate adaptation between model and LSO neurons (Fig 4F, black trace) and led to lowered overall spike counts during DRA (Fig 4G, solid lines). This effect of slow gain control had little effect on the overall amount of spatial information (Fig 4H), again indicative that the shift in ILD sensitivity in the LSO can be mostly explained by DRA in its monaural inputs (presumably already in the ANFs, compare also S3 Fig for analysis of additional stimulus paradigms). Nonetheless, addition of a slow gain control specifically increased D/spike of model responses for ILDs from the concurrent HPR (Fig 4I; compare dotted and solid lines). These modeling results thus suggest that the main function of slow gain control in the LSO is the optimization of coding efficiency (i.e., separability per unit of neuronal activity).

### Intrinsic imaging reveals energetic benefits of slow GABAergic gain control

**Fig 5:**
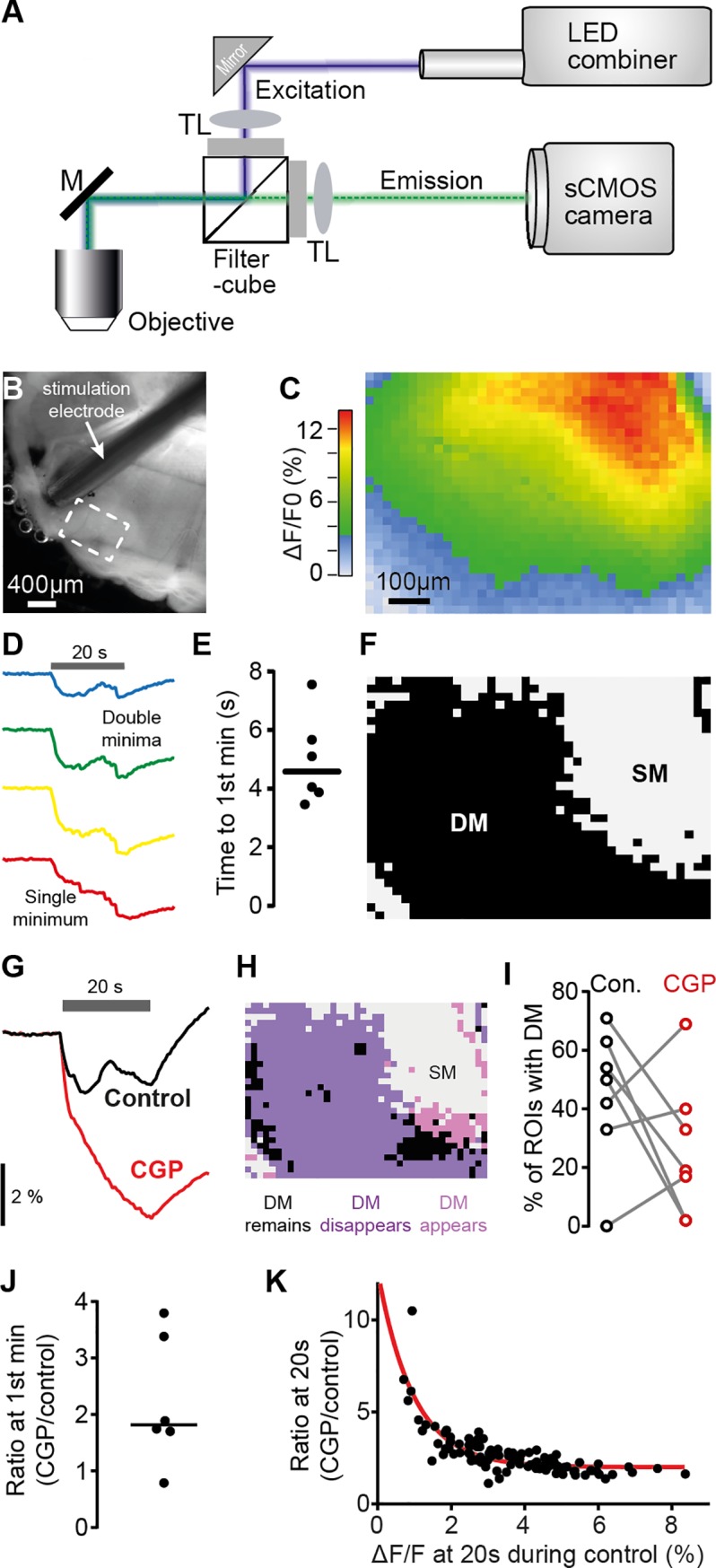
GABA-B signaling regulates NADH consumption in the LSO in an activity-dependent manner. (A) Schematic view of the optical path for intrinsic fluorescence imaging of metabolic activity in the LSO. (B) Bright-field image of brainstem (oblique illumination with IR-LED). Dashed rectangular denotes imaged area shown in (C). (C) Heat map illustrating spatial distribution of maximal relative decrease of NADH autofluorescence in the LSO in response to 20 s fiber stimulation at 200 Hz. (D) Exemplary traces for the temporal evolution of changes of NADH levels (colors correspond to respective regions in C). Gray horizontal bar denotes duration of electrical stimulation. While only an SM was present in the bottom trace (red, corresponding to large NADH decrease), the top three traces with less overall NADH decrease exhibited DM. (E) Mean time from stimulation start to first minimum calculated for each slice from those ROIs that exhibited DM. Population median: 4.58 s. (F) Distribution of SM and DM in the recording shown in (C). (G) Exemplary CGP-induced change in NADH fluorescence of a single ROI. (H) Spatial distribution of CGP-induced changes of NADH response types (SM/DM) for the recording shown in (C). (I) CGP-induced changes in the fraction of ROIs showing DM (*n* = 6 slices). (J) The average CGP/control ratio of NADH levels at mean time point of first minima (independent of presence of first minimum in the respective ROIs) was 1.6. (K) CGP/control ratio of NADH levels as function of NADH level changes in the control measurement (both measured 20 s after onset, in 100 neighboring ROIs in C); red line corresponds to exponential fit (tau = 0.97%; Spearman correlation, r = 0.8176, *P* < 0.0001). Underlying data can be found in S1 Data. CGP, CGP 55845 hydrochloride; Con., control; DM, double minima; GABA, *gamma*-aminobutyric acid; IR, infrared; LED, light-emitting diode; LSO, Lateral Superior Olive; M, mirror; NADH, nicotinamide adenine dinucleotide; ROI, region of interest; sCMOS, scientific Complementary metal-oxide semiconductor; SM, single minimum; TL, tube lens.

To directly test the model prediction that slow feedback signaling may minimize energy expenditure in subpopulations of LSO neurons, we took advantage of the intrinsic autofluorescence of a key intermediate nicotinamide adenine dinucleotide (NADH) during metabolic activity [42,43]. Specifically, our rationale was to examine energy consumption across large regions of the LSO during prolonged activity in an in vitro brain slice preparation using a self-designed imaging system for determining the changes in relative levels of NADH [41] (see Materials and methods). This technique allowed us studying the temporal and spatial evolution of energy expenditure and testing of any energy-minimizing effect by gain-control mechanisms. To this end, we monitored the relative change in NADH levels with high spatial resolution in LSO brain slices (21 μm × 23 μm per region of interest [ROI], 1,200 ROIs per field of view; Fig 5A and 5B; see Materials and methods). Using 20-s–long fiber stimulation of the excitatory inputs to the LSO at 200 Hz, we determined the spatial distribution of energy consumption in the LSO (six brain slices). As expected, large parts of the imaged LSO area displayed a monotonic increase in energy consumption with a single minimum (SM) in response to the 20-s–long stimulation (Fig 5C, red region; Fig 5D, lowest trace). However, we also frequently observed areas in which energy consumption declined after a few seconds of stimulation before ultimately increasing again (Double minima [DM]; Fig 5D and 5F). This nonmonotonic progression of energy consumption, combined with its apparent slow time course (4.58 s; IQR: 2.36 s; Fig 5E), is highly suggestive of the known GABA-B-receptor–mediated, activity-dependent gain-control mechanism. Accordingly, application of the specific antagonist CGP 55845 hydrochloride (CGP, 10 μM) to the bath revealed that DM largely disappeared during blockade of GABA-B signaling, resulting in considerably larger energy consumption (Fig 5G). In accordance with the assumed gain-control function of GABA, on the population level (i.e., across all ROIs per slice), CGP had differential effects on the prevalence of observed DM. A spatial diversity in the effect of blocking GABA-B mediated inhibition was clearly observable within individual brain slices (Fig 5H). Specifically, DM were either more or less likely to appear during CGP dependent on the fraction of DM during control (Fig 5I). Application of CGP also had a striking effect on the overall energy consumption in the LSO: across the entire imaged area, the block of GABA-B signaling on average almost doubled the energy consumption (median CGP/control ratio: 1.6, IQR: 1.5; *n* = 6 slices; Fig 5J). Moreover, similar to the history-dependency observed for the DMs, the magnitude of change in the energy consumption during CGP application was highly correlated with the prior activity level during control conditions (Spearman correlation, *P* < 0.0001; Fig 5K), providing further corroboration for the activity dependency of the gain-control mechanism. Together, these data strongly suggest that the spatially variable, slow gain control mediated by GABA-B in the LSO serves for the efficient population coding of ILDs.

## Discussion

Our findings advocate a novel, to our knowledge, concept for the neuronal detection and primary encoding of spatial cues. We observed that LSO neurons strongly adapted their ILD rate functions in response to changes in the input statistics. Consequently, ILD representation is dynamic and devoid of absolute mapping of sound-source locations already on the detector level. We further discovered that the average rate tuning of single LSO neurons conveys little spatial information during complex stimulation because of high response variability. However, if responses to individual instances of an ILD were averaged across neurons, DRA optimized the efficiency of responses, which resulted in improved separation of ILDs from the concurrent HPRs. Correspondingly, human listeners showed evidence of a focal improvement in ILD resolution specifically for HPR ILDs. Importantly, this study is—to our knowledge—the first to demonstrate stimulus-specific benefits by DRA both for the efficiency of neuronal coding as well as human perception. Finally, a simple LSO model and intrinsic energy imaging explained that the efficiency of the enhancement in spatial separability is facilitated by a slow gain-control mechanism involving GABAergic signaling downstream to binaural integration.

The established concept of spatial encoding assumes that specific average response rates of sensory neurons are mapped onto a particular physical cue to allow for a faithful encoding of the corresponding source location [19–21]. A recent study by Dahmen and colleagues [15] was instructive in suggesting that this assumption does not unrestrictedly hold because they found that already in the midbrain, ILD sensitivity was modulated by stimulus statistics, thus promoting a relative coding of sound-source positions. We expanded this concept and determined a prominent role of DRA for binaural processing that refutes the idea of an absolute representation of space already on the detector level. In this sense, our study represents an extension to earlier reports that had established the susceptibility of spatial tuning to adaptation by stimulus history [44–48].

However, since these earlier studies were conducted in downstream targets of the primary spatial cue detectors, the site of modulation was unresolved. We determined that the observed shifts in ILD sensitivity in the LSO are predominantly generated by the combinatory effect of upstream monaural adaptation to absolute sound level in the excitatory and inhibitory pathways (most likely the ANFs). This finding is not only crucial for the assessment of the functional role of DRA in the LSO (see next paragraph) but also bears significance towards the interpretation of previous finding of adaptive ILD coding at downstream processing stages such as the midbrain [15]. The fact that already in the LSO, DRA of single cells was not ILD-specific (i.e., not beneficial for the separability of adjacent ILDs within the HPRs) strongly indicates that monaural adaptational mechanisms are the dominant driver for shifts in ILD sensitivity at any processing stage. Thus, fundamentally, our data highlight the importance of considering monaural DRA to sound level (as control data) when assessing the specificity of adaptation in binaural neuronal sensitivity along the auditory pathway.

Our evaluation of the impact of DRA on neuronal information further suggests that the basic principle of LSO spatial coding is the preservation of ecologically relevant coding efficiency by providing high separability of nearby sound sources within the statistically predominant range of ILDs [5,40]. In accordance with this interpretation, recent studies reported modulatory effects of a preceding stimulus (adapter) on perceptual spatial resolution, both for ILDs and the other binaural cue, the interaural time difference (ITD) [49–51]. These findings corroborate the generality of stimulus-history–dependent effects on spatial perception. Importantly, it has been shown that the improvements in JND cannot be explained by unspecific attentional effects because acoustic priming of spatial attention to the location of the probe via the adapter by itself does not alter JNDs. Rather, it requires a congruence of both spectral content and the relevant binaural cue between adapter and probe to elicit an improvement in spatial resolution [49,51]. These findings clearly demonstrate that JND improvements are caused by adaptation in the spatial processing circuits in the respective frequency channels and not by attentional priming. Our present data and a prior study on ITD processing (33) provide a mechanistic explanation on the detector level that has so far been linked to secondary processing at higher stages [13,15,49,52,53].

Because the LSO represents the initial binaural stage of ILD detection, our findings stand out for two more reasons: (I) Adaptive processing at the spatial cue detector should result in absolute localization errors because of a missing reference frame. This notion is supported by reports of human listeners producing significant absolute localization errors when presented with biased spatial statistics [15,33,50,52,54]. (II) While adaptation with the purpose to preserve a large dynamic coding range within the predominant stimulus range can be found across sensory systems [7], we showed that in the LSO, DRA is likely to be inherited to a large degree by adaptation to intensity statistics in the monaural inputs (e.g., the auditory nerves from each ear). This susceptibility to differences in adaptation between the two inputs due to different input statistics largely explained the observed DRA in the LSO (compare Fig 4 and S1 Fig) and may also explain findings of LSO sensitivity to overall intensity [55]. We furthermore show that the major computational modification after binaural integration serves to optimize the efficiency of coding for the concurrent ILDs by further decreasing population spike rates. Such stimulus-statistic–specific processing to maximize the efficiency of information transmission (by redundancy reduction) has so far been associated with the midbrain and cortex, i.e., processing that is secondary to the initial detection of the respective feature [56–60]. In contrast, ILD detection and efficiency optimization are realized concurrently by the LSO (and subsequent negative feedback; see below). Interestingly, adaptation to binaural statistics to optimize spatial sensitivity has also been described for the detector neurons of the second important binaural cue, the ITDs [28,61]. However, in contrast to the short-term changes of the LSO, these adaptations take place over days during maturation and entail long-term morphological changes.

Different from prior studies on adaptation to spatial statistics in the midbrain [12,15], MLE(mean) declined for the concurrent HPR ILDs because of the high response variability of individual neurons. An informational gain was only revealed by applying a single-observation pseudopopulation coding concept in the form of MLE(pop). In this regard, our data provide physiological support for the framework of cooperative population decoding [38], which has been developed to explain the apparent noisiness of cortical processing. Specifically, the framework suggested that recurrent inhibition with a slow time constant can be utilized to maximize the efficiency of an average population code at the expense of increased response variability of individual neurons. Congruent with such a coding regime, individual LSO neurons responded sparsely (intermitted and with few spikes) and therefore decreased the redundancy of firing in the population for a given ILD. A potential limitation for such an interpretation of our data is that the neurons were not recorded at the same time (because of methodological limitations for brainstem recordings of highly stimulus-time–locked responses) and thus bear the possibility of overestimating the population advantage because of missing noise correlations. However, it is known that spiking in auditory brainstem nuclei occurs independently [62], and our in vitro recording of a large population of LSO neurons conclusively supports the single-neuron data. Accordingly, population analyses of single-neuron recordings are assumed a valid approximation and thus are commonly performed [12,13,15].

In conclusion, our findings suggest a new concept for spatial coding in the LSO: the detection and processing of ILDs is optimized for efficient sound-source separation in a given stimulus context by sparse population coding. This coding regime not only is energy efficient but also allows for detecting changes in the auditory periphery during high activity levels (noisy conditions) by maintaining high resolution. In ecologically plausible situations, the accompanying detriment of absolute localization accuracy might be compensated by an orienting head movement to bring the sound source into frontal space.

## Materials and methods

All data underlying the presented quantitative observations can be found in S1 Data.

### Ethics statement

Animal experiments using ketamine/xylazine anesthesia were approved by the German animal welfare act (District Government of Upper Bavaria, reference number: 55.2-1-54-2531-105-10). Psychophysical testing with human subjects (data were analyzed anonymously) was approved by the Ethics Committee of the Medical Faculty of the LMU (59–16).

### Electrophysiology

In vivo extracellular single-cell recordings were made from the LSO of young adult (postnatal age >80 days, *n* = 7 animals) Mongolian gerbils (*Meriones unguiculatus*) of both sexes.

To anesthetize the animals, a combination of ketamine (Ketavet, 100 mg/mL; Pfizer Inc., New York, NY, USA) and xylazine (Xylazin, 100 mg/mL; Sigma-Aldrich Chemie GmbH, Munich, Germany) was used. Physiological sodium chloride solution (NaCl, 0.9%; B. Braun Medicare GmbH, Melsungen, Germany) was mixed with 20% ketamine and 2% xylazine. After weighing the animals, they were anesthetized with an intraperitoneal injection (0.5 ml per 100 g body weight) of this anesthetic. After initial injection, the anesthetic was continuously provided by an automatic pump (801 Syringe Pump; Univentor High Precision Instruments Ltd., Zejtun, Malta) at a rate of 1.6 to 2.8 μl per minute depending on body weight and state of anesthesia. The anesthetic stage was periodically tested with the hind leg reflex. Constant body temperature of 37°C was ensured and checked by a thermostatically controlled heating pad the animals were placed on (Harvard Homeothermic Blanket Control Unit Model #50–7129; Harvard Apparatus Inc., Holliston, MA, USA). In order to ensure a sealed placement of the headphones on the acoustic meatus, the tragus was cut at two sides. The pericranium was anesthetized with lidocaine (Xylocain Pumpspray dental; AstraZeneca GmbH, Wedel, Germany).

A small cut of the skin was made across the rostrocaudal axis on the upper part of the skull, and a craniotomy and a durotomy (ca. 1.5 × 2.5 mm) approximately 1,800 mm lateral to the midline and 4,500 mm caudal to the bregmoid axis was performed. Ringer solution was periodically applied to the opening to prevent damage of the brain surface due to dehydration. The animals’ body functions were monitored though various devices. The heart rate and breathing cycle was monitored optically and acoustically through an electrocardiogram. The animals’ blood oxygen was measured through a pulse oximetry monitor (LifeSense Tabletop Capnography and Pulse Oximetry Monitor; Nonin Medical Inc., Plymouth, MN, USA). The animal was also typically provided carbogen through a custom-made mask. Recording sessions typically lasted between 10 to 12 hours.

The recording site was marked by iontophoretic application of the enzyme horseradish peroxidase (HRP). Experiments were then finalized by euthanizing the animals without awakening by an intraperitoneal injection of 1 ml of 20 mg/ml pentobarbital in Ringer solution. The animals were transcardially perfused with Ringer solution and 4% paraformaldehyde (PFA) for approximately 30 minutes. The brain was carefully removed from the cranium and put into 4% PFA at 4°C upon further processing to determine the recording location. Only recordings from locations that were positively identified within the LSO were used for further data analysis.

Extracellular single-cell recordings were obtained using pulled glass micropipettes (1.5 mm OD × 0.86 mm ID, GC150F-10; Harvard Apparatus Ltd) filled with 1 M HRP in 1 M NaCl and a resistance of 7 to 10 MΩ (measured with Ωmega-Tip Z; World Precision Instruments Inc., Sarasota, FL, USA). The electrode was mounted on a piezo drive (Inchworm controller 8200; Burleigh Products Group Inc., Victor, NY, USA), which was connected to a motorized manipulator (Digimatic series 164 type 161; Mitutoyo Deutschland GmbH, Neuss, Germany). The electrode signal was amplified (Electro 705, World Precision Instruments Inc. and Wide Band Amplifier, TOE 7607; Toellner GmbH, Herdecke, Germany) and fed to a computer via an A/D-converter (TDT RP2.1, System III; Tucker-Davis Technologies Inc., Alachua, FL, USA), where the signal was filtered. A notch filter was used to filter the 50-Hz electrical noise caused by the power line hum, and a high-pass filter with 300 Hz and low-pass with 5-kHz bandpass filtered the signal in the RP2.1. Brainware (Jan Schnupp, University of Oxford, Oxford, UK, for Tucker-Davis Technologies Inc., USA) was used to visualize and analyze incoming spike trains. The spike times and raw traces were recorded and saved for subsequent analysis.

### Stimulus generation and presentation

All the stimuli were digitally generated using MATLAB (The MathWorks Inc., Natick, MA, USA) and fed into TDT hardware using Brainware. The stimuli were D/A-converted in a TDT Multi-Function Processor (TDT RX6, System III; Tucker-Davis Technologies Inc.) and then attenuated with a TDT Programmable Attenuator (TDT PA5, System III; Tucker-Davis Technologies Inc.). The analog signal was delivered to the headphones. To cover the wide range of the LSO’s frequency spectrum, either Etymotic Research headphones ER-10B+ with ER 10D-T04 silicon ear tips (Etymotic Research, Inc., Elk Grove Village, IL, USA) or custom-build electrostatic headphones were used. The same silicon ear tips were fitted to either headphones to have a comparable seal to the animals’ ears. Custom-written calibration filters were used to achieve a flat spectrum over the entire range of the respective headphones.

When spikes of single cells were identifiable, the characteristic frequency (CF) and absolute threshold were determined using a pure-tone stimulus having the same length as the search stimulus. For further characterization of a neuron, a baseline ILD function was obtained and a broadband noise rate-level functions were recorded in response to 50-ms bursts presented on the ipsilateral excitatory ear only. These recordings were used for determining a cell’s latency (median latency = 4.2 ms, IQR = 3.5 ms, *n* = 25 cells).

To measure DRA in LSO neurons, a bimodal HPR stimulus was created. The intensity of continuous broadband noise was drawn from a pseudorandomized predefined distribution every 50 ms (see Fig 1). The range of monaural intensities spread from 20 to 80 dB SPL in 2 dB steps, similar as used monaurally [16,17]. The predefined distribution consisted of two HPRs’ intensity levels around center intensities of 50 dB ± 4 dB SPL and 70 dB ± 4 dB SPL, resulting in 5 values per HPR with a cumulative occurrence probability of 0.8. To generate ILDs, the stimulus intensities were mirrored at 60 dB for presentation on the other ear, resulting in HPR center regions of −20 dB ILD and +20 dB ILD, respectively.

A single condition epoch was 6.55 seconds long and was repeated 10 times with different pseudorandomizations each time (but identical cumulative probabilities). A 131-s–long stimulus was generated by alternating the two HPR conditions repeatedly, resulting in 10 HPR epochs per sweep for each condition. The stimulus sequence was identical across recordings. Acquisition of a complete set of stimuli typically lasted >2 h, thereby making the acquisition of a large sample size challenging.

### Neuronal data analysis

Recorded data files were analyzed offline using custom-made analysis in MATLAB and Python. First, the average latency of each cell was determined on the basis of its monaural rate-level functions to allow for subsequent spike-triggered analysis of responses to the HPR stimuli. To this end, spikes were assigned to 50-ms bins of the respective ILD that elicited the spikes (taking into account the latency of the cell). This resulted in a mean ILD response-rate function for each HPR condition.

Spike-triggered stimulus averages where calculated to investigate whether a particular ILD sequence influenced the spiking if neurons. To this end, we selected all bins with a nonzero response and determined the ILD values presented during this time bin and during the nine previous bins. We then averaged the determined ILD values for each of the 10 bins. The data are plotted relative to the mean ILD of the bin that triggered the spike. The non-spike–triggered average was calculated the same way but based (i.e., triggered) on bins that did not show any response.

The standard separation D is calculated as previously described [36]:

$$D_{n}=\left|mu_{n}+1‐mu_{n}\right|/\left(sqrt\left(sigma_{n+1}\;*\;sigma_{n}\right)\right),$$

where mu_n + 1_ and mu_n_ are the mean values of the responses to two ILD values while sigma_n + 1_ and sigma_n_ are their standard deviation. D_n_ was subsequently smoothed using a 5-sample moving average filter.

The metric D/spike was calculated as

$$D_{n},spike=\left(2\;*D_{n}\right)/\left(mu_{n+1}+mu_{n}\right).$$

In the case of the model, we calculated D based on the assumption of an underlying Poisson process in which the variance would equal the mean response.

MLEs were used to find the most probable ILD to result in a specific observed response *R*_*obs*_ given all other observed responses *R*. For this, the joint probability density functions *P*(*R*, *ILD*) of the observed spike counts *R* and the presented ILDs were calculated for all responses of one neuron, excluding *R*_*obs*_. The ILD that maximizes *P*(*R* = *R*_*obs*_, *ILD*) was then used as the MLE for *R*_*obs*_.

To characterize shifts in ILD functions due to HPR statistics, the threshold ILD—defined as the ILD at which the firing rate differentiates more than 10% from baseline firing—was determined.

Time courses of adaptation were measured by fitting a single- or double-exponential function to the mean responses rates averaged over all 30 repetitions of an HPR condition. Inbuilt functions in MATLAB for the rmse and the adjusted coefficient of determination (R^2^) were used to evaluate the goodness of fits.

### Psychophysical measurements and data analysis

Five normal-hearing (within 20 dB of ISO/TR 389–5:1998) listeners (2 males and 3 females, mean age 26 ± 4 years, right-handed) participated in the measurement of just-noticeable ILD differences. The signals consisted of white noise that was generated in MATLAB at a sampling rate of 44.1 kHz. The signals were digital to analog converted (Audio 2 Dj; Native Instruments GmbH, Berlin, Germany) before being presented over circumaural headphones (HDA 200; Sennheiser Electronic GmbH & Co. KG., Wedemark, Germany), which were calibrated for a flat frequency response between 20 Hz and 20 kHz. The signals were presented at 60-dB SPL average diotic sound-pressure level, and ILDs were introduced by symmetrically amplifying and attenuating the right and left ear signals by half the desired ILD. Within the experiment, a 2-s–long adapter stimulus was followed, after 350 ms, by two 50-ms probe stimuli that were separated by 100 ms. Similar to the physiological experiments, the adapter consisted of concatenated diotic noise bursts, each 50 ms in duration, with ILDs that were randomly drawn from one of the two nonuniform HPR distributions.

ILD JNDs were determined at two reference ILDs (i.e., −20 dB ILD and +20 dB ILD). One of the two probe stimuli was randomly presented at one of the two reference ILDs, while the other probe stimulus was systematically varied using a transformed up–down procedure, following a one-up three-down rule, as implemented by the MATLAB AFC package [63]. To determine the JND, listeners were asked to specify the perceived direction of the probe pair sounds, which allows deducing which of the two probe stimuli was perceived as more lateralized. Following the subject’s answer, the variable probe ILD was adjusted until reaching the termination criterion (6 reversals) of the one-up three-down rule. ILD JNDs for each listener, each probe position, and each listening condition (i.e., HPR) were calculated as median over six sessions (each session consisting of 3 measurements). For each subject, the effect of listening condition was expressed as normalized change in ILD JND because of colocation of probe position and preceding HPR.

### LSO model

The LSO was modeled using a phenomenological rate model similar to the one used by Wen and colleagues [17] to model adaptation in the ANF (see S1 Fig). The LSO is implemented as a subtraction stage with inputs from the ipsi- and contralateral ANFs and a sigmoidal activation function (CN and MNTB were omitted to minimize model complexity). The firing rate *R*_*LSO*_(*t*) in spikes per second (sps) of the LSO is calculated as follows:

$$R_{LSO}(t)=\frac{R_{max}-R_{min}}{1+e^{-k(R_{diff}(t)-R_{0})}}+R_{min}$$

$$R_{diff}(t)=R_{ipsi}(t)-g∙R_{contra}(t),$$

where *R*_*max*_ and *R*_*min*_ are the maximum and the minimum firing rates, *R*_0_ is the rate at zero input, and *k* is the steepness of the sigmoid. *R*_*ipsi*_(*t*) and *R*_*contra*_(*t*) are the firing rates from the ipsi- and the contralateral ANFs, and *g* is a gain factor to weight the relative strength of the excitatory and inhibitory inputs. The ANF inputs were each calculated using a dual adaptation model [17], which was fitted to the data shown in Fig 2 of [16]. Because we only fitted the response of one ANF, we switched the saturating nonlinearity used in the original model with a simple logistic function.

The parameters for the LSO model were determined by calculating the ILD rate function of the model and fitting it to the ILD rate function given by Fig 4 in [64] (resulting parameters: $R_{max}=200sps,k=0.19\frac{1}{sps},R=42sps,g=0.69$). The slow LSO adaptation was implemented to resemble the second adaptation stage of [17]. The adaptation parameters where adjusted so that the time course of adaptation was in agreement with the recorded data from the LSO. This resulted in an adaptation time constant of 4 ms, *g*_1_ = 0.3, *g*_2_ = 0.01, with an adaptation threshold of 35 sps (see [17] for details on the implementation). Running this model often resulted in zero firing rates for larger negative ILD values, which led to undefined D/spike values, so we introduced a minimal LSO firing rate of *R*_*min*_ = 30 sps.

### LSO intrinsic metabolic imaging

Changes in NADH levels in the LSO were monitored by imaging of NADH autofluorescence in acute brainstem slices as recently described [43,65]. The animals were anesthetized with isoflurane and decapitated. We removed the brains and cut 250-μm–thick transverse slices (VT1200S Vibratome; Leica Microsystems GmbH, Wetzlar, Germany). The slices were superfused at room temperature (22–25°C) in the recording chamber with gassed (95% O_2_ and 5% CO_2_) artificial cerebrospinal fluid (ACSF) solution containing (in mm): 23 sucrose, 125 NaCl, 25 NaHCO_3_, 2.5 KCl, 1.25 NaH_2_PO4, 1 MgCl_2_, 2 CaCl_2_, 2 glucose (Sigma-Aldrich). NADH was excited with a 365-nm LED, and fluorescence images (emission filter: 447 ± 30 nm) were recorded at 2 Hz (pco.edge 5.5; PCO AG, Kehlheim, Germany).

LSO neurons were electrically excited by a 20-s stimulation train at 200 Hz with biphasic pulses of 1 ms duration and 5-V amplitude using a bipolar Tungsten electrode placed in the fiber tract targeting the LSO. NADH fluorescence intensity was measured in individual rectangular ROIs, corrected for photobleaching, and presented as ΔF/F0 (F0 = fluorescence level at stimulation onset; ΔF = change in fluorescence level relative to F0).

The occurrence of DM was automatically detected based on their amplitudes (>0.05–0.10%), the time difference between them (>6–10 s), and the time differences between the minima and the interjacent maximum (>1–2 s). These parameters were individually adjusted for each slice by analyzing a measurement with larger ROIs and by comparing the automated results with those of visual inspection of the individual traces. Specific blockade of GABA-B receptors was performed by application of 10 μM CGP [(2*S*)-3-[[(1*S*)-1-(3,4-Dichlorophenyl)ethyl]amino-2-hydroxypropyl](phenylmethyl)phosphinic acid hydrochloride] (Tocris Bioscience, Bristol, UK) to the ACSF for 20 minutes.

## Supporting information

S1 Fig Schematics of additional HPR stimulus paradigms. (A, F, and K) The intensity distribution on the ipsilateral ear was identical to the main HPR paradigm (Fig 1) but altered on the contralateral ear (A and F) or not stimulated at all (ipsi only, K). For (A) and (F), the intensity distribution on the contralateral ear was fixed for an entire epoch at 70 dB or 50 dB and switched between HPR conditions. These intensities were either presented out of phase with the mean HPR intensity on the ipsilateral ear (contra paradigm 2, panel A) or in phase (contra paradigm 3, panel F). (B, G, and L) Mean ILD response functions of all LSO neurons tested with the respective paradigm. Conventions as in Fig 2C. (C, H, and M) Scatter plot of threshold ILDs illustrates significant changes with HPR condition in paradigm 2 (*P* = 0.0003, *N* = 18 neurons, paired Wilcoxon signed-rank test) and paradigm 3 (*P* = 0.0005, *N* = 19 neurons, paired Wilcoxon signed-rank test) but not for the ipsi-only paradigm (*P* = 0.4, *N* = 11 neurons, paired Wilcoxon signed-rank test). Conventions as in Fig 1. (D–E and I–J) Similar to the original stimulus paradigm (Fig 1 and Fig 3), the advantage of these shifts for ILD computation is displayed when calculating D(pop) but not for D(mean) analysis. In contrast to Fig 3D, the asymmetry of the stimulus paradigms 2 and 3 do not allow calculating summed D-functions across both hemispheres. Note that the peaks of D(pop) are very close to the respective HPRs but do not perfectly align. In contrast, D(mean) takes minimal values within the respective HPRs in either condition. Underlying data can be found in S1 Data. HPR, high-probability region; ILD, interaural level difference; LSO, Lateral Superior Olive.
(TIF)

S2 Fig (A) Spike-count histograms for four representative LSO neurons (aligned in rows) to a full epoch (approximately 6.55 s) in each HPR condition (left column: −20 dB HPR condition, right column: +20 dB HPR condition). Bins (i.e., 50-ms snippets) during which the ILDs took the center value of the respective HPR in each condition (−20 dB and +20 dB, respectively) are color-coded, illustrating a high response variability. (B) Histogram of Pearson correlations of LSO responses to the full HPR stimulus. The average correlation of spike counts across three repetitions of the entire stimulus set (19 switches) was surprisingly low for the majority of neurons (median Pearson correlation coefficient = 0.62, IQR: 0.26). (C) A spike-triggered analysis of the responses of all neurons established that the likelihood of spiking to any ILD was not systematically associated with a prior occurrence of specific relative ILDs (upper panel; color code represents HPR conditions). Performing the same analysis but triggered by nonspiking to a represented ILD (lower panel) exposed a tendency of nonresponsiveness due to presentation of a more positive ILD shortly before. (D) The mean Pearson correlation coefficients of responses to the same ILDs was significantly higher when stimuli were presented with gaps of 300 ms in between (“Control”) compared to either HPR condition (paired-sample *t* test, *N* = 13 neurons). Underlying data can be found in S1 Data. HPR, high-probability region; ILD, interaural level difference; IQR, interquartile range; LSO, Lateral Superior Olive.
(TIF)

S3 Fig (A) Comparison of goodness of fits for power law and exponential fitting of the adaptation time course in LSO neurons. Left: Rmses using a power-law fit were lower compared to a single-exponential fit (left, *P* = 0.001, Wilcoxon signed-rank test), but slightly higher compared to a double-exponential fit (right, *P* = 0.02, Wilcoxon signed-rank test). (B) Block diagram of the LSO rate model. The input to the model is given as a sequence of sound levels on the ipsi- (L_ipsi_) and contralateral (L_contra_) ear. A dual adaptation model is used to calculate the ANF firing rates R_ipsi and R_contra. The LSO model is implemented as a subtraction stage, with the contralateral input weighted by a gain value and a following sigmoid to model the activation of the neuron. An optional adaptation stage that resembles the rate adaptation stage in the ANF model is used to account for the slow adaptation component present in the LSO measurements. (C) Model responses (left panel) and resulting D(pop) in response to contra paradigm 2 (see S1 Fig). The model closely captures both the magnitude of ILD tuning function shifts and corresponding changes in D(pop) (compare S1 Fig). (D) Same as in (C), but for contra paradigm 3 (compare S1 Fig). Underlying data can be found in S1 Data. ANF, auditory nerve fiber; ILD, interaural level difference; LSO, Lateral Superior Olive; rmse, root mean-squared error.
(TIF)

S1 Data Primary data set. This file contains all individual numerical values used to generate figures in this manuscript.
(XLSX)

## Abbreviations

ACSF: artificial cerebrospinal fluid
ANF: auditory nerve fiber
CF: characteristic frequency
CGP: CGP 55845 hydrochloride
CN: Cochlear Nucleus
DM: double minima
DRA: dynamic range adaptation
GABA: *gamma*-aminobutyric acid
HPR: high-probability region
HRP: horseradish peroxide
ILD: interaural level difference
IQR: interquartile range
IR: infrared
ITD: interaural time difference
JND: just-noticeable difference
LED: light-emitting diode
LSO: Lateral Superior Olive
MLE: Maximum Likelihood Estimation
MNTB: Medial Nucleus of the Trapezoid Body
NADH: nicotinamide adenine dinucleotide
PFA: paraformaldehyde
rmse: root mean-squared error
ROI: region of interest
sCMOS: scientific Complementary metal-oxide semiconductor
SM: single minimum
sps: spikes per second
