# Supplementary figures and images for: Cooperative population coding facilitates efficient sound-source separability by adaptation to input statistics
Source: PLoS Biol. 2019 Jul 29;17(7):e3000150. doi: 10.1371/journal.pbio.3000150 (PMC6687189; doi:10.1371/journal.pbio.3000150)

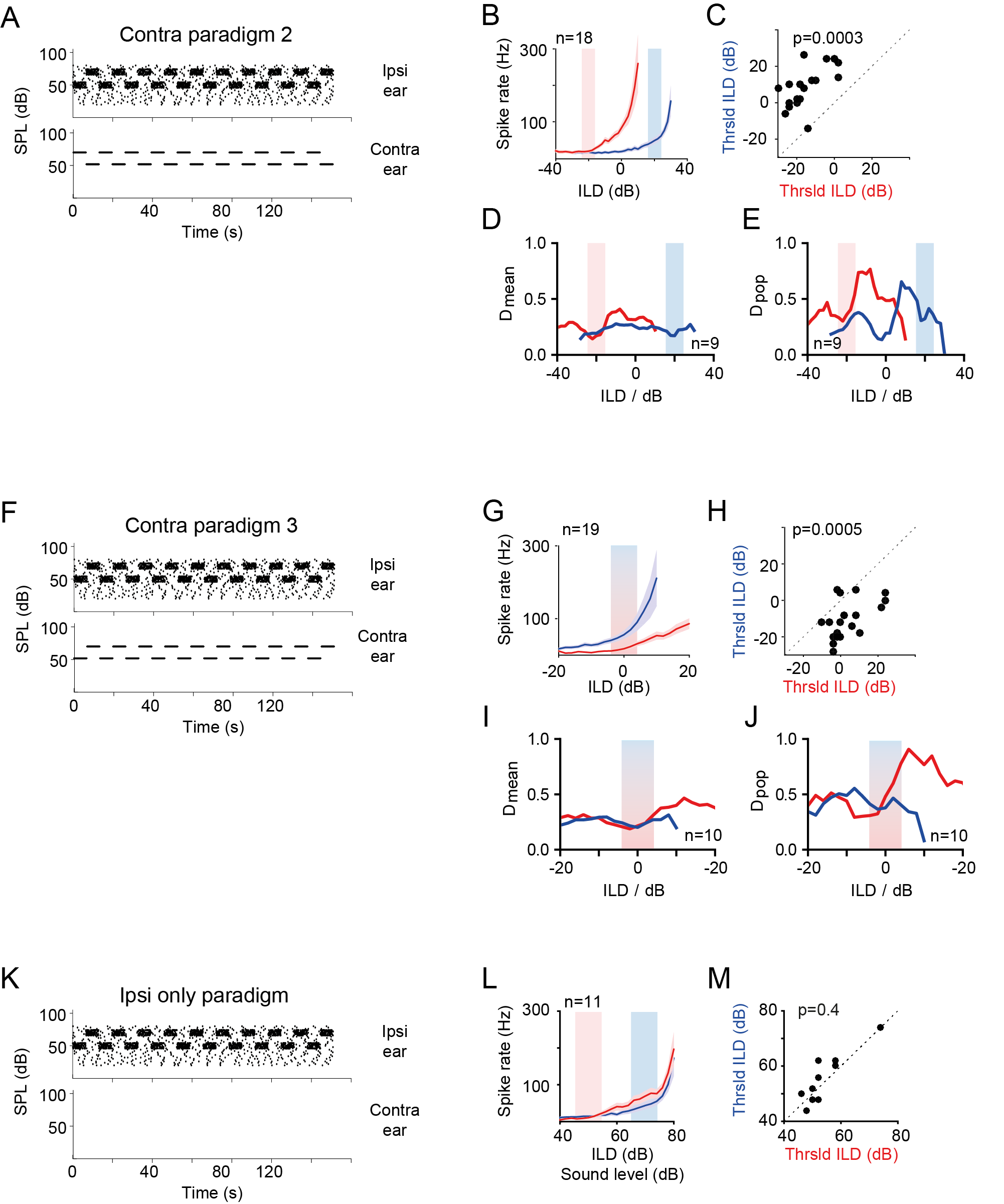

Supplement: S1 Fig — (A, F, and K) The intensity distribution on the ipsilateral ear was identical to the main HPR paradigm (Fig 1) but altered on the contralateral ear (A and F) or not stimulated at all (ipsi only, K). For (A) and (F), the intensity distribution on the contralateral ear was fixed for an entire epoch at 70 dB or 50 dB and switched between HPR conditions. These intensities were either presented out of phase with the mean HPR intensity on the ipsilateral ear (contra paradigm 2, panel A) or in phase (contra paradigm 3, panel F). (B, G, and L) Mean ILD response functions of all LSO neurons tested with the respective paradigm. Conventions as in Fig 2C. (C, H, and M) Scatter plot of threshold ILDs illustrates significant changes with HPR condition in paradigm 2 (P = 0.0003, N = 18 neurons, paired Wilcoxon signed-rank test) and paradigm 3 (P = 0.0005, N = 19 neurons, paired Wilcoxon signed-rank test) but not for the ipsi-only paradigm (P = 0.4, N = 11 neurons, paired Wilcoxon signed-rank test). Conventions as in Fig 1. (D–E and I–J) Similar to the original stimulus paradigm (Fig 1 and Fig 3), the advantage of these shifts for ILD computation is displayed when calculating D(pop) but not for D(mean) analysis. In contrast to Fig 3D, the asymmetry of the stimulus paradigms 2 and 3 do not allow calculating summed D-functions across both hemispheres. Note that the peaks of D(pop) are very close to the respective HPRs but do not perfectly align. In contrast, D(mean) takes minimal values within the respective HPRs in either condition. Underlying data can be found in S1 Data. HPR, high-probability region; ILD, interaural level difference; LSO, Lateral Superior Olive. (TIF) [file pbio.3000150.s001.tif]

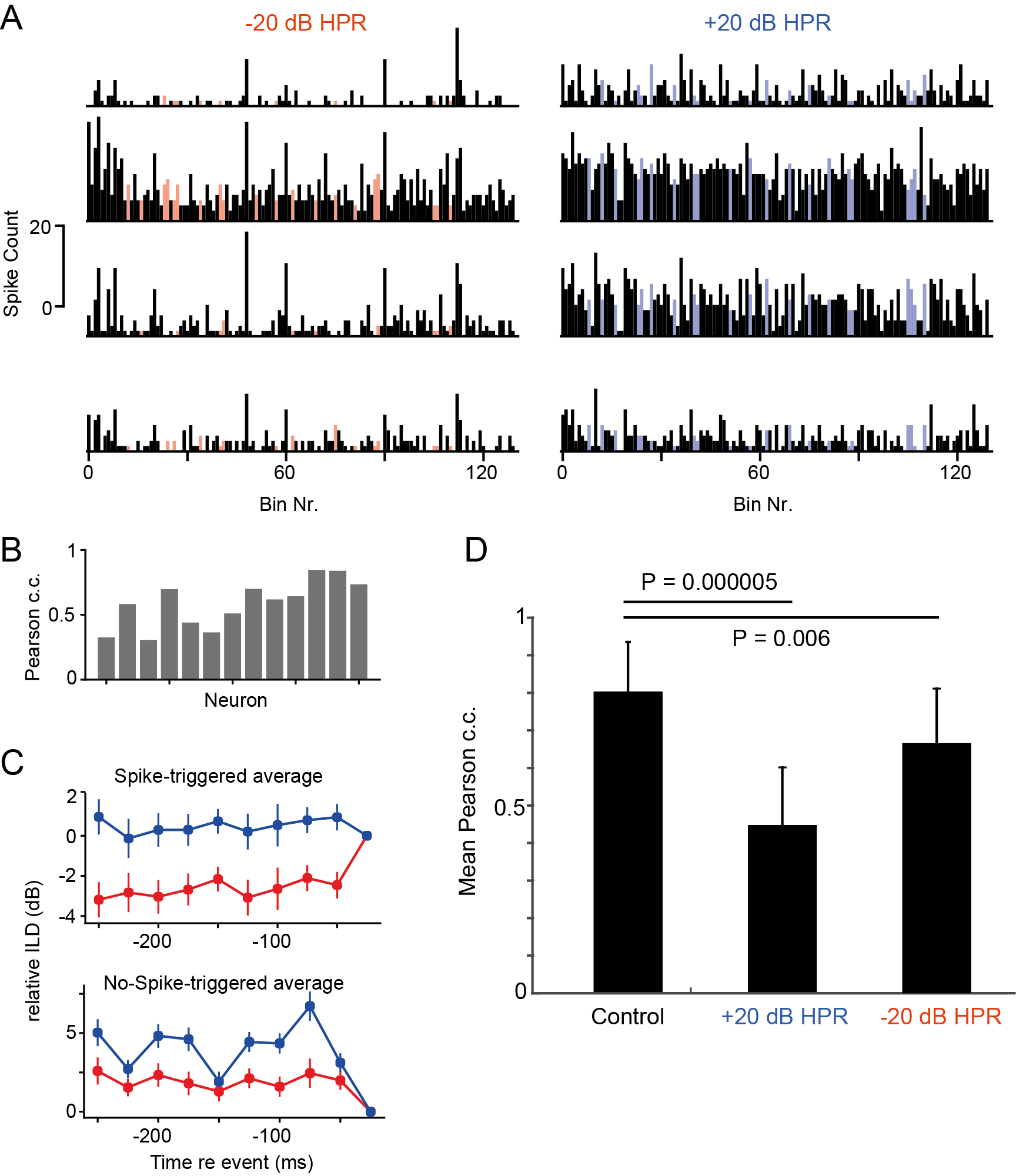

Supplement: S2 Fig — (A) Spike-count histograms for four representative LSO neurons (aligned in rows) to a full epoch (approximately 6.55 s) in each HPR condition (left column: −20 dB HPR condition, right column: +20 dB HPR condition). Bins (i.e., 50-ms snippets) during which the ILDs took the center value of the respective HPR in each condition (−20 dB and +20 dB, respectively) are color-coded, illustrating a high response variability. (B) Histogram of Pearson correlations of LSO responses to the full HPR stimulus. The average correlation of spike counts across three repetitions of the entire stimulus set (19 switches) was surprisingly low for the majority of neurons (median Pearson correlation coefficient = 0.62, IQR: 0.26). (C) A spike-triggered analysis of the responses of all neurons established that the likelihood of spiking to any ILD was not systematically associated with a prior occurrence of specific relative ILDs (upper panel; color code represents HPR conditions). Performing the same analysis but triggered by nonspiking to a represented ILD (lower panel) exposed a tendency of nonresponsiveness due to presentation of a more positive ILD shortly before. (D) The mean Pearson correlation coefficients of responses to the same ILDs was significantly higher when stimuli were presented with gaps of 300 ms in between (“Control”) compared to either HPR condition (paired-sample t test, N = 13 neurons). Underlying data can be found in S1 Data. HPR, high-probability region; ILD, interaural level difference; IQR, interquartile range; LSO, Lateral Superior Olive. (TIF) [file pbio.3000150.s002.tif]

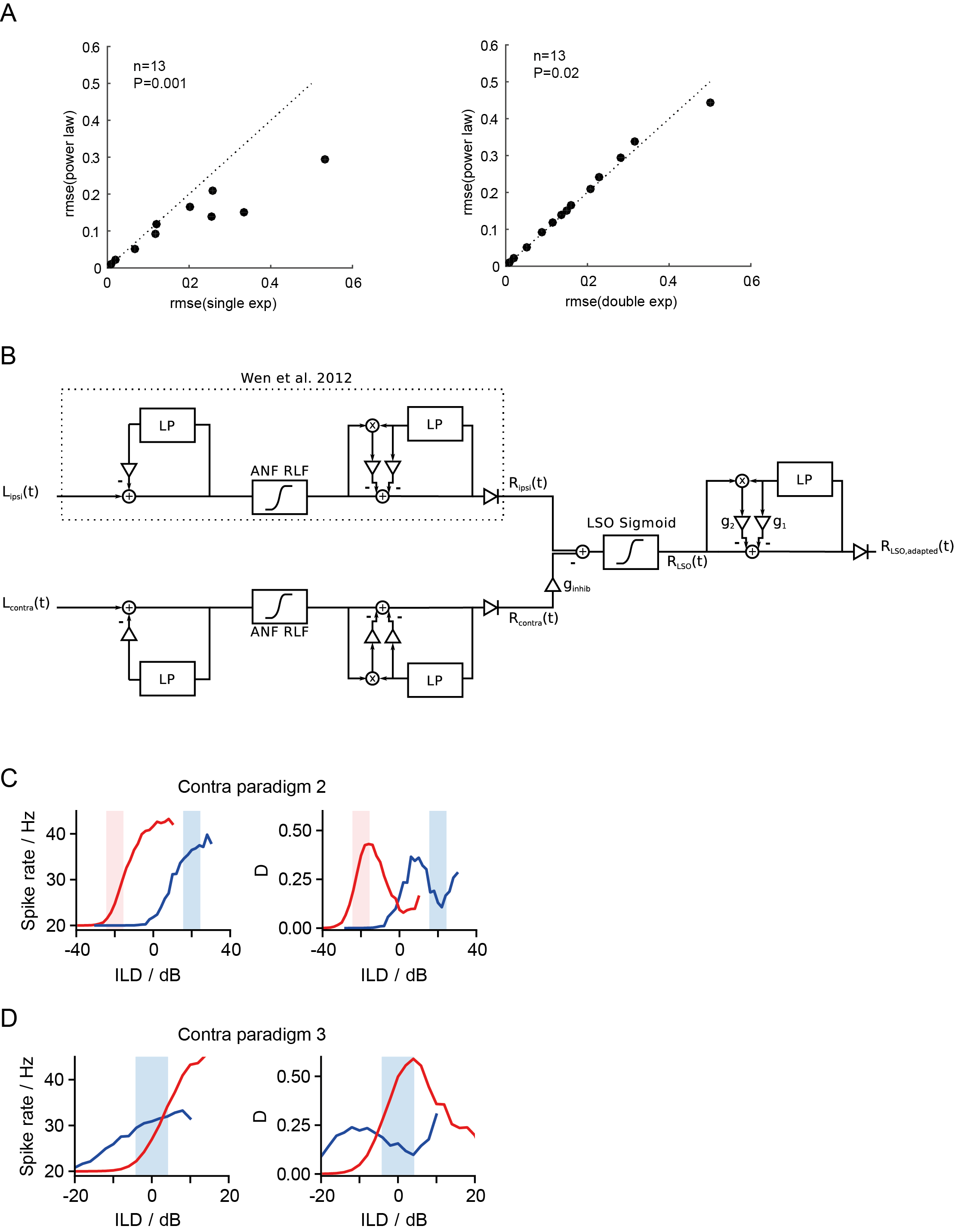

Supplement: S3 Fig — (A) Comparison of goodness of fits for power law and exponential fitting of the adaptation time course in LSO neurons. Left: Rmses using a power-law fit were lower compared to a single-exponential fit (left, P = 0.001, Wilcoxon signed-rank test), but slightly higher compared to a double-exponential fit (right, P = 0.02, Wilcoxon signed-rank test). (B) Block diagram of the LSO rate model. The input to the model is given as a sequence of sound levels on the ipsi- (Lipsi) and contralateral (Lcontra) ear. A dual adaptation model is used to calculate the ANF firing rates R_ipsi and R_contra. The LSO model is implemented as a subtraction stage, with the contralateral input weighted by a gain value and a following sigmoid to model the activation of the neuron. An optional adaptation stage that resembles the rate adaptation stage in the ANF model is used to account for the slow adaptation component present in the LSO measurements. (C) Model responses (left panel) and resulting D(pop) in response to contra paradigm 2 (see S1 Fig). The model closely captures both the magnitude of ILD tuning function shifts and corresponding changes in D(pop) (compare S1 Fig). (D) Same as in (C), but for contra paradigm 3 (compare S1 Fig). Underlying data can be found in S1 Data. ANF, auditory nerve fiber; ILD, interaural level difference; LSO, Lateral Superior Olive; rmse, root mean-squared error. (TIF) [file pbio.3000150.s003.tif]
